# Three-Dimensional Leaf Edge Reconstruction Combining Two- and Three-Dimensional Approaches

**DOI:** 10.34133/plantphenomics.0181

**Published:** 2024-05-09

**Authors:** Hidekazu Murata, Koji Noshita

**Affiliations:** ^1^ Department of Biology, Kyushu University, Fukuoka, Fukuoka 819–0395, Japan.; ^2^ Plant Frontier Research Center, Kyushu University, Fukuoka, Fukuoka 819–0395, Japan.

## Abstract

Leaves, crucial for plant physiology, exhibit various morphological traits that meet diverse functional needs. Traditional leaf morphology quantification, largely 2-dimensional (2D), has not fully captured the 3-dimensional (3D) aspects of leaf function. Despite improvements in 3D data acquisition, accurately depicting leaf morphologies, particularly at the edges, is difficult. This study proposes a method for 3D leaf edge reconstruction, combining 2D image segmentation with curve-based 3D reconstruction. Utilizing deep-learning-based instance segmentation for 2D edge detection, structure from motion for estimation of camera positions and orientations, leaf correspondence identification for matching leaves among images, and curve-based 3D reconstruction for estimating 3D curve fragments, the method assembles 3D curve fragments into a leaf edge model through B-spline curve fitting. The method's performances were evaluated on both virtual and actual leaves, and the results indicated that small leaves and high camera noise pose greater challenges to reconstruction. We developed guidelines for setting a reliability threshold for curve fragments, considering factors occlusion, leaf size, the number of images, and camera error; the number of images had a lesser impact on this threshold compared to others. The method was effective for lobed leaves and leaves with fewer than 4 holes. However, challenges still existed when dealing with morphologies exhibiting highly local variations, such as serrations. This nondestructive approach to 3D leaf edge reconstruction marks an advancement in the quantitative analysis of plant morphology. It is a promising way to capture whole-plant architecture by combining 2D and 3D phenotyping approaches adapted to the target anatomical structures.

## Introduction

Leaves are highly important organs for plants since they are the sites of fundamental physiological processes, including photosynthesis, transpiration, and respiration. The phenotypic diversity of leaves underlies the various functional demands associated with their habitats [1–4]. Furthermore, their morphological properties are essential in balancing the multiple functional demands of individual plants and canopies [5–7], such as light interception [8,9], heat transfer [10,11], hydraulic conductivity [12,13], mechanical constraints [14,15], and growth efficiency [16,17]. Quantifying the morphological traits of leaves provides a quantitative understanding of the relationships between the morphological traits and genetics of plants, morphogenesis, and environmental conditions, providing valuable insights into plant growth and development, improving crop yields, and enhancing plant productivity.

Leaves have complex 3-dimensional (3D) shapes. Despite this, traditional measurement, quantification, and evaluation techniques rely on 2-dimensional (2D) methods because they are simple and more feasible to use, especially considering existing technical limitations. In many cases, botanical specimens are preserved 2-dimensionally [18] and undergo morphological changes upon drying [19,20]. Quantitative evaluations are based on 2D imaging (e.g., flatbed scanners) and image analysis (e.g., [21,22]). Leaves exhibit a wide range of patterns in 3D shapes [23], and their functionality is highly dependent on their configuration in 3D space [24–27]. For example, the spatial configurations and 3D shapes of leaves affect light interception and penetration within individual plants [28] and canopies [29]. These 3D leaf shapes also contribute to light and heat acclimatization (e.g., lamina folding [29,30] and nonplaner leaves [30,31]). Therefore, the 3D shape of leaves is crucial for agricultural applications, with its impact on photosynthesis at the canopy level being investigated in major crops such as maize, wheat, and rice through the development of morphological models and evaluation techniques [32–35]. According to the studies incorporating simulations with morphological and growth models, such as functional-structural plant models (FSPMs), accounting for the 3D leaf structure may influence the conditions necessary for optimal plant growth [36,37]. In regulating such functional leaf shapes through morphogenesis, the marginal region of the leaf, including leaf edges, is crucial, serving as a place for integrating mechanical properties, genetic controls, differentiation patterns, and tissue growth [38]. Moreover, some shapes cannot be adequately projected 2-dimensionally (e.g., twisted leaves of *Codiaeum variegatum* ‘*Spirale*’). Consequently, many leaf characteristics have not been appropriately evaluated through 2D methods, inspiring interest in 3D evaluations.

High-resolution 3D morphological data can be acquired efficiently and cost-effectively using light detection and ranging sensors, depth cameras, and photogrammetry techniques [39–41]. A pipeline utilizing structure from motion (SfM) and multiview stereo (MVS), which reconstructs a 3D surface as point cloud data from a series of 2D images captured from different angles, has been implemented in several libraries and software products (e.g., [42,43]). Several devices and techniques for acquiring the structures of plants in 3D have been developed to facilitate 3D evaluation in plant phenotyping studies [44,45]. However, point cloud data produced by point-based 3D reconstruction methods, such as the commonly used SfM/MVS pipeline, may not be ideal for representing 3D leaf morphologies because of unclear leaf edges [46] and uncertainties regarding whether the holes in point cloud data are actually real or the results of reconstruction errors [47]. Point cloud data reconstructed using the point-based reconstruction method often include points representing both leaves and artifacts owing to the keypoints detected in the background (Fig. S1A). Even if the background regions are excluded by using the mask images, the inherent nature of being represented as a set of points makes it challenging to recognize the exact position of the leaf edges. The holes in the output point cloud data comprise reconstruction deficiencies and actual holes; it is difficult to distinguish between them solely based on point cloud data (Fig. S1B and C). It is preferable to establish phenotyping methods that enable the direct estimation of leaf edges.

**Fig. 1.**
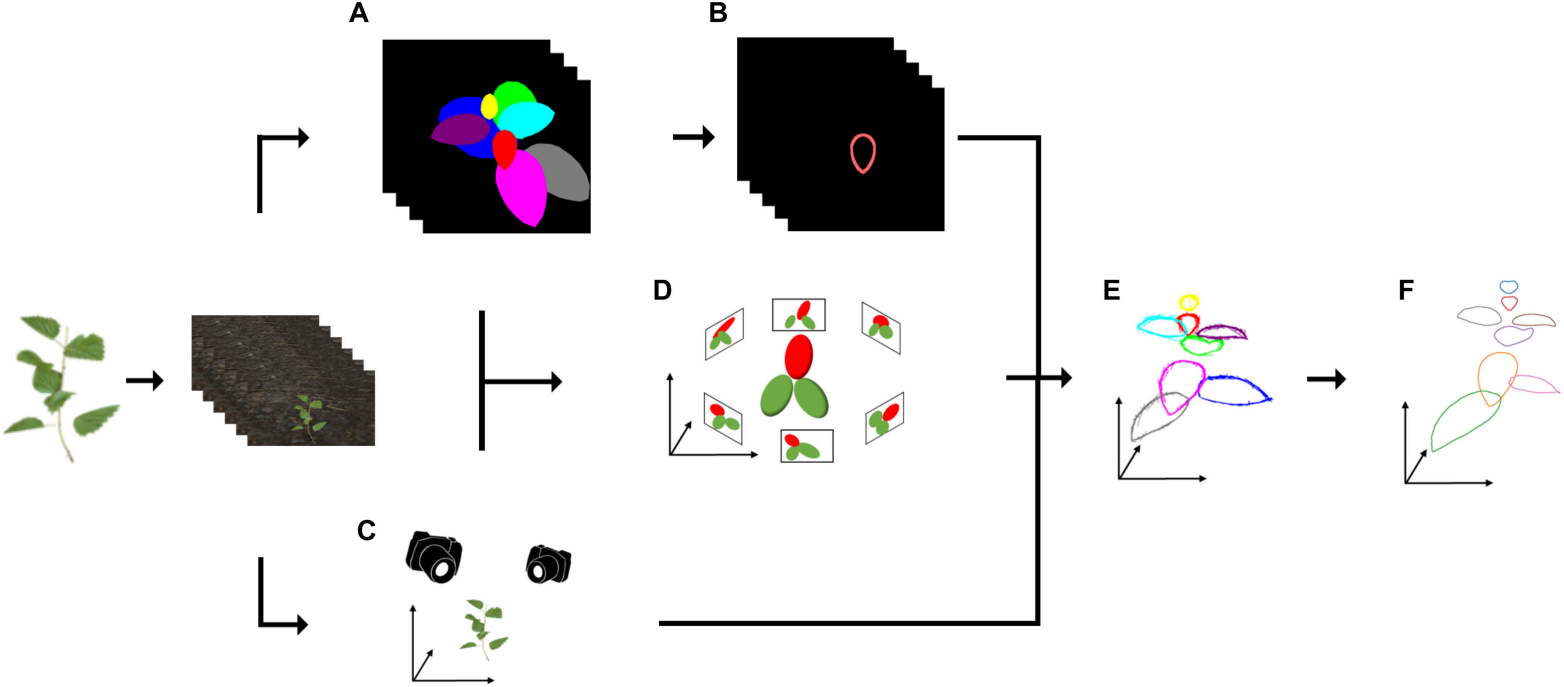
Overview of the proposed method for 3D leaf edge reconstruction. The method reconstructs 3D leaf edges from multiview images. (A) Each leaf in each image is segmented using Mask R-CNN. (B) Each 2D leaf edge is detected from the segmented leaves. (C) Camera positions and orientations are estimated based on SfM. Simultaneously, sparse point cloud data and projection matrix are obtained for the leaf correspondence step, in which (D) the leaves in the multiview images are identified. (E) The curve fragments are reconstructed in 3D space using the 3D curve sketch, which integrates the 2D leaf edges, projection matrix, and leaf correspondence. (F) The 3D leaf edges are obtained after fitting closed B-spline curves on each set of 3D curve fragments corresponding to a single leaf.

In this study, we proposed a method to reconstruct leaf edges from multiview images using deep-learning-based instance segmentation for 2D edge detection (Fig. 1A and B), SfM for estimating camera positions and orientations (Fig. 1C), leaf correspondence identification for matching leaves among multiview images (Fig. 1D), curve-based 3D reconstruction for estimating leaf edges as curve fragments in 3D spaces (Fig. 1E), and B-spline curve fitting for integrating curved fragments into 3D leaf outlines (Fig. 1F). The applicability and limitations of the proposed method were examined using both simulated data and actual multiview images of soybean plants. Our analysis revealed that leaf size, errors in camera parameter estimation, and mask estimation errors had significantly impacted accuracy. The proposed method is expected to be a valuable tool for clarifying the morphological characteristics of 3D leaf edges, which are difficult to quantitatively evaluate.

## Materials and Methods

### A method for 3D leaf edge reconstruction using a combination of 2D and 3D approaches

To estimate leaf edge morphological properties directly in 3D Euclidean space, we proposed a method to reconstruct 3D leaf edges from multiview images. We assumed that the multiview images were obtained from the simple photogrammetry system (Fig. S2). Then, the 3D leaf edges are reconstructed via the following procedure (Fig. 1):

#### Instance segmentation of leaves in 2D images

To extract the 2D edges of leaves individually, mask images for each leaf were obtained from multiview images (Fig. 1A) using Mask R-CNN [48], a deep neural network (DNN) model for instance segmentation. We used Detectoron2 [49], a library for detection and segmentation tasks, to utilize the Mask R-CNN model with the backbone ImageNet and the model weights pretrained on the COCO dataset. The model was trained on a training dataset that comprised 80% of the dataset consisting of multiview images, and the remaining 20% of images were used for validation (validation dataset) (see Actual data for details).

#### Leaf edge extraction in 2D images

Leaf edges in the 2D images were extracted from the predicted mask image for each instance (Fig. 1B), using the OpenCV library [42]. The extracted 2D edges were divided into fragments that have a certain range of lengths (*l*_min_, *l*_max_) and minimum overlap length *τ*_overlap_ for utilizing the curve-based 3D reconstruction (see [50] for details). In this study, we used *l*_min_ = 40 pixels, *l*_max_ = 100 pixels, and *τ*_overlap_ = 15 pixels for the simulated data and *l*_min_ = 80 pixels, *l*_max_ = 200 pixels, and *τ*_overlap_ = 30 pixels for the real data, depending on their image sizes (see Materials).

#### SfM

The SfM technique was utilized to obtain the projection matrix for each camera and the sparse point cloud from a multiview image (Fig. 1C). SfM is a photogrammetric method for simultaneously estimating the camera parameters and the depth of corresponding points (i.e., sparse 3D point clouds) from multiview images. In this study, we used Metashape (Agisoft, St. Petersburg, Russia), which is commercial photogrammetry software that includes SfM. The projection matrices, including the optical center, focal length, orientation, and position of the cameras, were exported as Extensible Markup Language (XML) files. Markers were placed on the image to optimize image placement and thereby make it easier to obtain the corresponding points.

#### Leaf correspondence identification

**Fig. 2.**
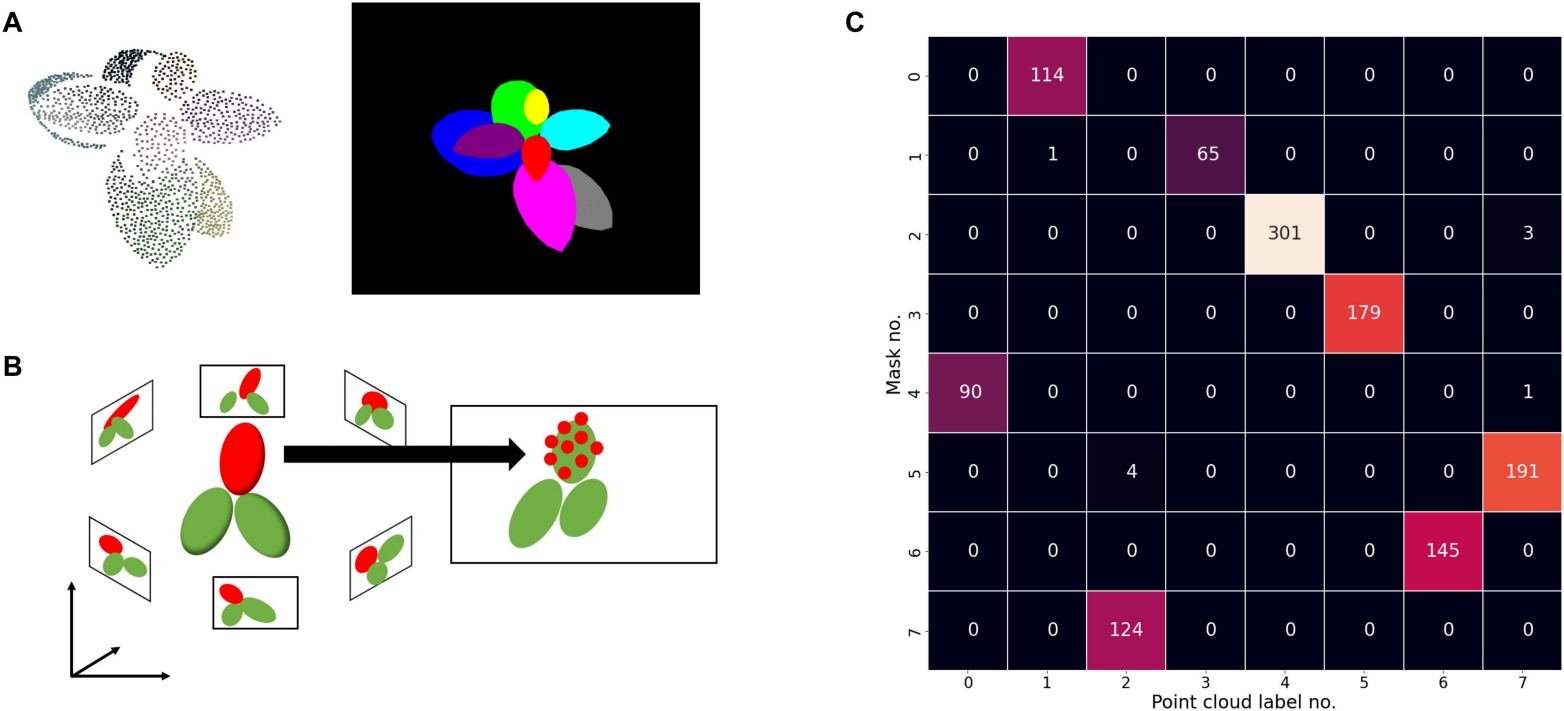
Leaf correspondence identification. (A) An example of a set of point cloud data clustered into each leaf with the hidden point removal from a particular viewpoint (left) and mask image of the corresponding view (right). (B) Correspondence of leaves between images is identified by projecting the clustered point cloud onto each image. (C) Heatmap of the count data of projected point cloud data on a mask image. Peaks indicating the correspondence between clusters and instances in a mask image.

To individually process and reconstruct the leaves, we determined the correspondence of the leaves between the images (Fig. 1D). First, the point cloud obtained from SfM was clustered into each leaf, i.e., each cluster corresponds to a single leaf (Fig. 2A). To preclude the leakage of points from the backside into the front during reprojection, hidden point removal [51] was applied to each view. Then, the point cloud was associated with the mask on which most of the points had been located (Fig. 2B). Leaf correspondences were identified by counting the number of reprojected points belonging to each cluster in each image (Fig. 2C). If this was performed for all the mask images, the correspondence between the leaves of the images could be obtained via a point cloud.

**Fig. 3.**
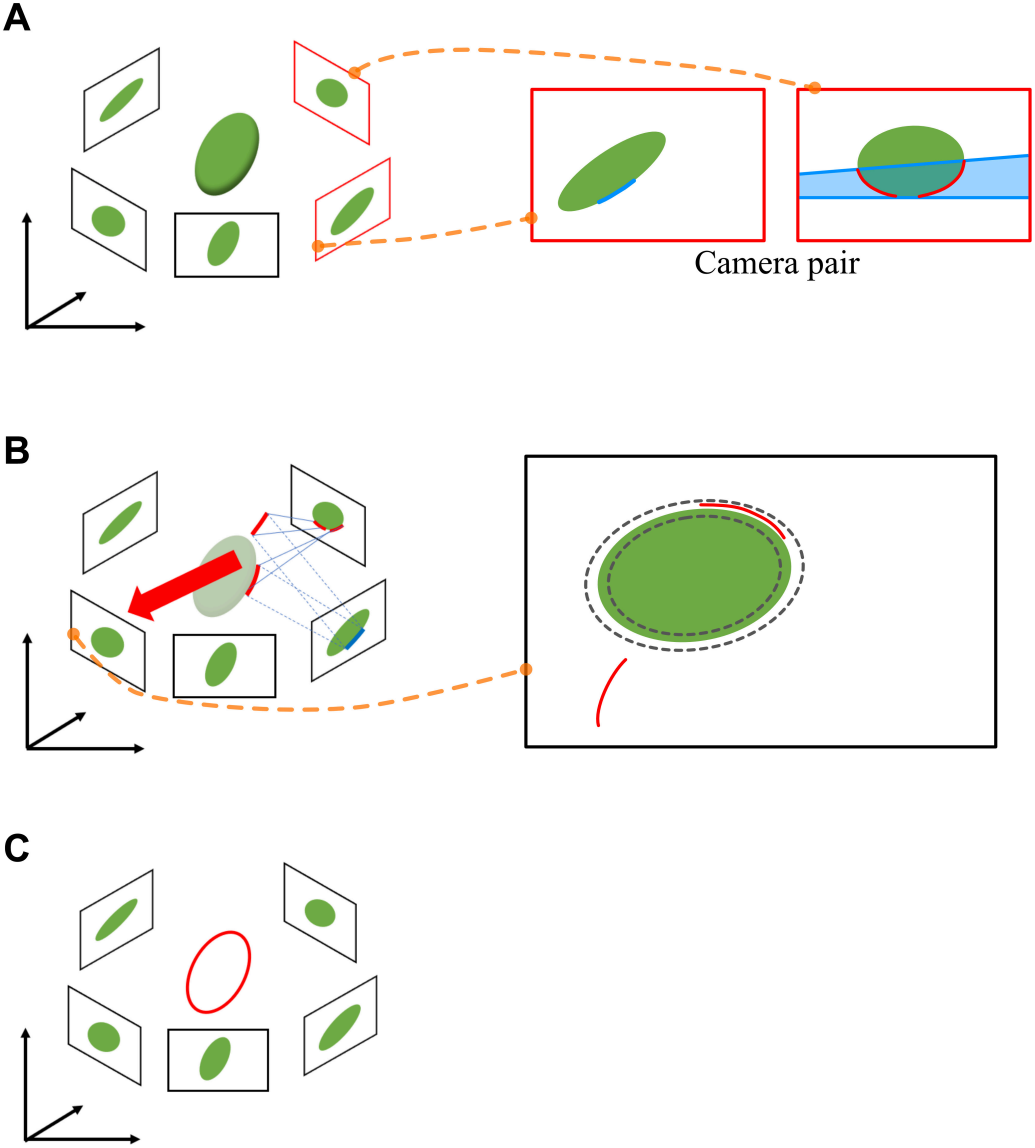
Curve-based 3D reconstruction of a leaf edge. (A) Pair hypotheses are generated in a camera pair by searching for intersecting curve fragments in the 2D images along a band of epipolar lines (blue band). (B) The 3D curve fragments are reconstructed and reprojected onto other images to evaluate how closely the reconstructed curve resembles the true projection. The pair hypothesis is supported if the reprojected 2D curve fragment sufficiently close to the 2D leaf edges (within gray dashed curves). (C) Only the 3D curved fragments supported by a sufficient number of images are reconstructed.

In this study, density-based spatial clustering of applications with noise (DBSCAN) [52] was used for clustering on simulated data. Color-based region-growing segmentation implemented in the Point Cloud Library [53] was used on real data because it is difficult to separate leaves in physical contact using DBSCAN. Hidden point removal [51], which determines the visible points in a point cloud from a given viewpoint using a sphere and a spherical inversion operator, was used for removing behind points.

#### Curve-based 3D reconstruction

The key idea of the proposed method is directly estimating 3D leaf edges using curve-based 3D reconstruction. In this study, we adopted a curve-based MVS reconstruction used in the work of Fabbri and Kimia [50], which proposed a method called 3D curve sketch that reconstructed a set of 3D curve fragments from the 2D edges of a target object in multiview images (Fig. 1E). All the subsequent processes were applied to each leaf. Obtaining 3D curve edges involves the following steps: (1) camera pair definition, (2) pair hypothesis generation, and (3) 3D curve fragment reconstruction and filtering by reprojection.

1. Camera pair definition: To perform curve-based 3D reconstruction, camera pairs were defined based on the relative positions of the cameras in the scene. Angle *b_ij_*, which is the angle between cameras *i* and *j* from the average positions of all the cameras ($\bar{p}$), was calculated for all the camera combinations. The camera pairs were defined as the combinations that satisfied *b_ij_* ≤ *b*_max_. Since the cameras had been assumed to be equally spaced to cover the plants, *b* corresponded to the baseline in [50]. In this study, we used angles of 30°, 40°, and 60° on the simulated data of 32, 64, and 128 multiview images, respectively. For the real data, *b*_max_ was set to 30°, regardless of the number of images.

2. Pair hypothesis generation: Let $\gamma_{p}^{i}$ be the *p*-th 2D curve fragment in the *i*-th image. A potentially corresponding pair of 2D curve fragments, called pair hypothesis, is defined as a pair of 2D curve fragments $\left(\gamma_{p}^{i},,,\gamma_{q}^{j}\right)$. In epipolar geometry, a fundamental matrix **F***_ij_* computed from the projection matrices corresponding to images (**P***_i_* and **P***_j_*) maps a point in the *i*-th image to a line in the *j*-th image. The line mapped by the fundamental matrix is called the epipolar line (or epiline), and any existing corresponding points along the line are found. By extending this concept to a 2D curve fragment, **F***_ij_* maps a 2D curve fragment in the *i*-th image to a band (a set of epipolar lines) in the *j*-th image. Pair hypotheses were generated based on the 2D curve fragments overlapping the bands (Fig. 3A). For a robust reconstruction, 2D curved fragments tangential to the epipolar line were excluded from the process (see [50] for details). The number of pairs of hypotheses per band was set to a maximum of only 10 to account for the limited computational resources.

**Fig. 4.**
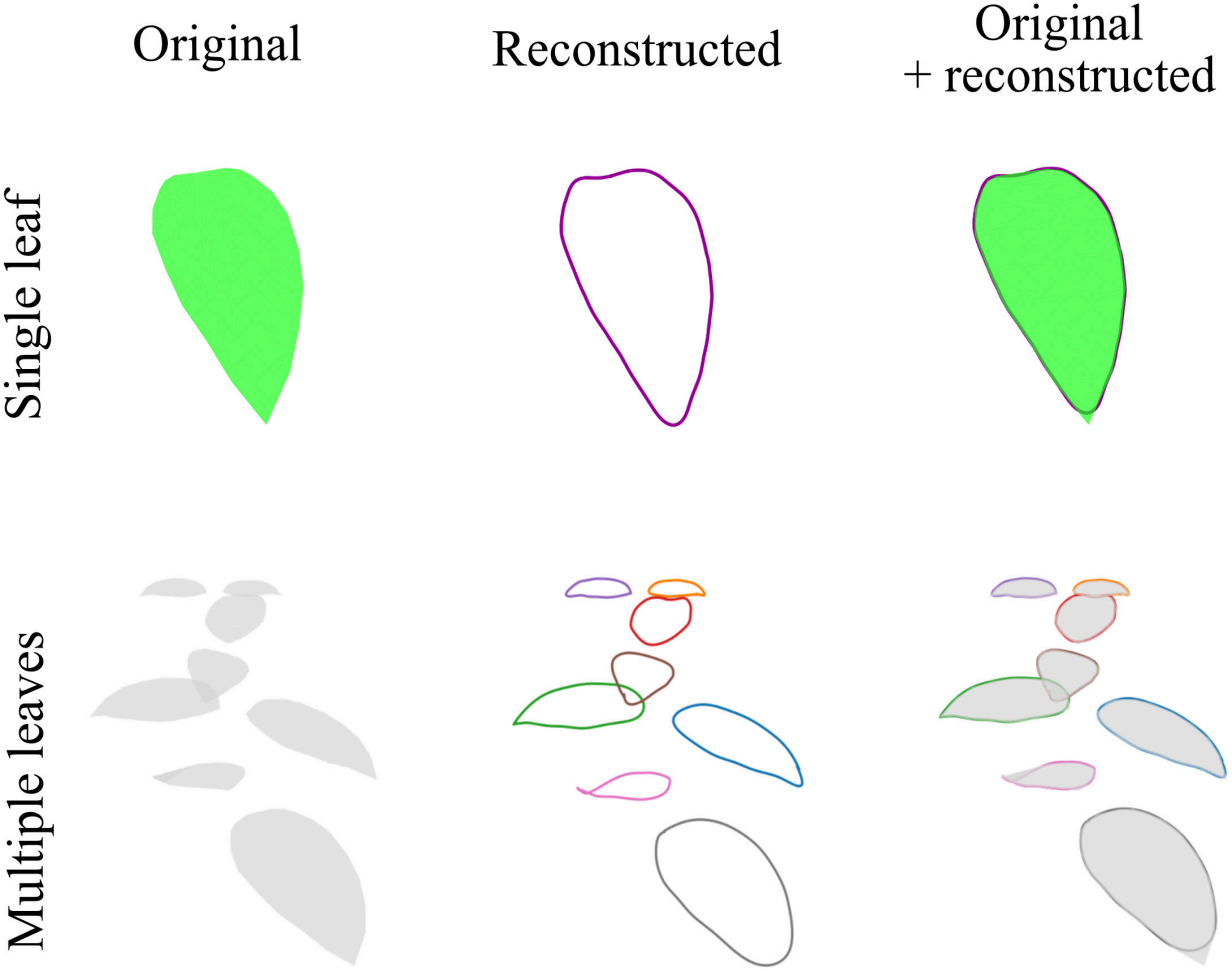
Examples of 3D leaf edge reconstruction on simulated leaves. Reconstructed 3D edges of a single leaf (upper, green) and multiple leaves (lower, gray) using the proposed method. Each reconstructed 3D leaf edge is indicated by a different color. Original meshes (left), reconstructed 3D edges (middle), and overlaid ones (right) are shown.

3. 3D curve fragment reconstruction and filtered by reprojection: Then, 3D curve fragments ($\Gamma_{p,q}^{i,j}$), which correspond to the pair hypotheses $\left(\gamma_{p}^{i},,,\gamma_{q}^{j}\right)$, were reconstructed using projection matrixes in 3D Euclidean space. Each reconstructed 3D curve fragment was reprojected onto multiview images, excluding the *i*- and *j*-th images, to evaluate how closely the reconstructed curve fragments generated the true projection (Fig. 3B). The reconstructed curve fragments were supported by reprojections if the reprojected curve fragments had been located close to the edges of the target object (i.e., leaf) on the image; i.e., a reprojected curve fragment was supported if at least *τ_v_* (%) of the curve fragment was located within *τ_d_* pixels of the edges in *τ_t_* images. Only curves supported with a sufficient number of images (i.e., greater than the support threshold *τ_t_*) were reconstructed (Fig. 3C). We also excluded points supported by less than *τ_p_* on a well-supported curve in addressing an issue related to the “erroneous grouping” described in the work of Usumezbas et al. [54], which proposed an enhanced method of Fabbri and Kimia [50]. A *τ_v_* of 80% was used for all cases, and *τ_d_* was 11 and 39 pixels for the simulated leaves and actual soybean specimens, respectively.

#### B-spline curve fitting

The 3D curve fragments were integrated into a closed 3D curve by using B-spline fitting (Fig. 1F). A B-spline function is a smooth piecewise degree *k* polynomial function. In the closed B-spline curve fitting, a continuous periodic function is approximated by the B-s, which is a linear combination of the order *j* B-spline basis over the *i*-th interval *b*_*i*, *j*_(*l*) as follows:

fl=wbl=w1⋯wn−1b1,jl⋮bn−k−1,jlbn−k,jl+b1−k,jl⋮bn−1,jl+b0,jl (1)

where *w_i_* denotes the coefficient of the *i*-th B-spline basis. Based on the coordinate values of the reconstructed 3D curve fragments, the B-spline coefficients were estimated for *x*-, *y*-, and *z*-coordinate values using the “curve_fit” function in SciPy [55]. In this study, the number of intervals (*n*) was set to 16 for all simulated and actual datasets (dataset_s1_, dataset_a1_, and dataset_a2_), with the exception of dataset_s2_, for which adopted *n* = 64.

### Materials

#### Simulated data

Virtual plant models (single and multiple leaves) were created using Blender (Blender Foundation, Amsterdam, Netherlands). Three individuals were generated based on the multiple-leaf model; each leaf was translated randomly—horizontally from −33.33 to 33.33% and vertically from −14.28% to 14.29% of the bounding box dimensions—and rotated randomly from −10 to 10°.

Based on the created models, we generated several multiview images from cylindrically arranged views using Unity (Unity Technologies, San Francisco, CA, US). The dataset (dataset_s1_) includes multiview images of various levels of occlusion (no, thin, and thick pillars), different numbers of multiview images (32, 64, and 128 images), and different degrees of positional noise affecting the camera parameters (*σ* = 0, 1, and 3 mm).

Moreover, we generated multiview images of 1,920 × 1,080 pixels from virtual single-leaf models, including a lobed leaf, a leaf with serration, elongated leaves, and leaves with holes (dataset_s2_). They were used to demonstrate the proposed method for complex leaf edges. The 3D models of a lobed leaf (“Maple Leaf” by Ciminera) [56] and a leaf with serration (“Leaf test” by Ivanovs) [57] are used under CC BY 4.0. The 3D models of leaves with holes were created using Blender.

#### Actual data

Multiview images were obtained from 4 individual soybeans (*Glycine max*), including 4 cultivars (Enrei, Zairai 51-2, Aoakimame, and Saga zairai), to train the Mask R-CNN model and evaluate its performance (dataset_a1_). These individuals were captured at different growth stages: Enrei: 34 days after sowing (DAS); Zairai 51-2: 56 DAS, Aoakimame: 24 DAS; and Saga zairai: 48 DAS.

To demonstrate the applicability of the proposed method, multiview images of another cultivar, Fukuyutaka, at different growth stages of 21, 28, and 42 DAS, were obtained (dataset_a2_). Each set of multiview images included 264 images, and approximately 130 images were subsampled.

These 5 soybean cultivars, which were included in the Japanese soybean mini-core collection [58], were obtained from the Genebank Project, NARO (National Agriculture and Food Research Organization).

To explore the potential applicability of the method to plants other than soybeans, multiview images of an individual of house plant (*Aglaonema* ‘Maria’) were captured, and each leaf was manually annotated (dataset_a3_). Using dataset_a3_, we attempted to reconstruct the 3D leaf edges based on the proposed method, excluding instance segmentation by Mask R-CNN.

We used a simple fixed photogrammetry system consisting of digital cameras (EOS Kiss X7; Canon, Tokyo, Japan), a turntable (MT320RL40; ComXim, Shenzhen, China), and a camera control application (CaptureGRID4; Kuvacode, Kerava, Finland) (Fig. S2) to obtain multiview images of 5,184 × 3,456 pixels.

### Testing the method to reconstruct 3D leaf edges

#### Accuracy of 3D leaf edge reconstruction

We evaluated the accuracy of the 3D leaf edge reconstruction method for different leaf areas, image numbers, occlusion levels, and noise levels on the dataset_s1_. This evaluation was performed on the simulated multiple-leaf data using the Fréchet distance [59] divided by the square root of the leaf area, hereinafter referred to as the standardized Fréchet distance (SFD). The SFD was calculated for 3 individual plants with 8 different-sized leaves (312 mm^2^ ≤ *A* ≤ 3,366 mm^2^) in several simulation scenarios, including different levels of occlusion (no, thin, and thick pillars), different numbers of multiview images (32, 64, and 128 images), and different degrees of positional noise affecting the camera parameters (*σ* = 0, 1, and 3 mm). The Mann–Whitney U test [60] with Bonferroni correction [61] was performed to investigate the differences in SFD among the different leaf area, positional error, and the number of images.

#### Optimization of the support thresholds

To obtain accurate 3D leaf edges, the support threshold (*τ*_t_) should be set appropriately to balance the trade-off between the number and precision of the reconstructed 3D curve fragments. We attempted to propose optimal support thresholds against occlusion indices (OIs) based on simulated virtual leaves by evaluating the precision-recall curve of the reconstructed 3D edges on the dataset_s1_. In this study, the OI of a target leaf was defined based on the sparse point cloud data of the target, as follows:

$$\text{OI}=\frac{1}{m}\sum_{i=1}^{m}\left(1-\frac{n_{i}}{n}\right)$$ (2)

where *m* is the number of images; *n* is the number of points of a target instance; *n_i_* is the number of points of a target instance reprojected onto the *i*-th image; and OI is the occlusion index, where OI = 0 indicates no occlusion, and OI = 1 indicates complete occlusion.

For *τ_t_*, the precision-recall curves in the ground-truth mesh and the reconstructed curve fragment were calculated in the simulation data. The optimal support threshold is the highest *τ_t_*, with the highest recall when the precision h exceeded 0.99; the precision is the percentage of ground truths for which the reconstructed curved fragments are within 30 mm, and the recall is the percentage of curved fragments for which the ground truths are within 30 mm. The simulation data were comprehensively tested for different precision and recall values with respect to the support threshold, which is defined as the ratio of image numbers to the total (from 0.125 to 1). If the precision did not reach one, the minimum value was used as the optimal support threshold. The Mann–Whitney U test [60] with Bonferroni correction [61] was subsequently performed to investigate the differences in the optimal support threshold among the different leaf area, positional error, and the number of images.

#### Confirmation of the proposed method on actual soybean data

Regarding the instance segmentation of the leaves using Mask R-CNN, the model performance was evaluated on dataset_a1_. To calculate the accuracy of instance segmentation using Mask R-CNN, we performed group 4-fold cross-validation, in which each group corresponds to multiview images of each individual. In each iteration, the model was trained on multiview image data of 3 individuals, split into training data (80%) and validation data (20%).

We demonstrated the performance of the proposed 3D leaf edge reconstruction method by applying it to individual soybeans (Fukuyutaka) at 3 growth stages (dataset_a2_). The 3D leaf edges were reconstructed using the support threshold proposed in the guidelines (Guidelines for setting support thresholds in 3D edge reconstruction).

#### Applicability of the proposed method for more diverse leaves

Using the proposed method, we attempted to reconstruct complex 3D leaf edges, which were challenging using point-based 3D reconstruction. To demonstrate this, we applied the proposed method to virtual leaves of dataset_s2_ (lobed leaf, leaf with serration, elongated leaves, and leaves with 1 to 6 holes) and actual leaves of dataset_a3_ (*Aglaonema* ‘Maria’). In the case of leaves with holes, DBSCAN was used to separate multiple holes and the leaf edge before the curve-based MVS reconstruction. Mask images corresponding to individual leaves in multiview images in dataset_a3_ were manually created, and the 3D leaf edges were reconstructed without the step of instance segmentation based on Mask R-CNN. In this study, the Mask R-CNN model was trained on dataset_a1_ consisting of only 4 soybean cultivars, and applying it to different plant species, crops, or cultivars requires training on a dataset tailored to them or a large dataset.

## Results

### Leaf edge reconstruction in 3D space on virtually generated leaf models

The proposed method was first demonstrated on virtual data generated from the models of single and multiple leaves under the ideal condition (i.e., specimens in dataset_s1_ with no pillars and no camera positional errors).

On single virtual leaves, true mask images and camera parameters are known. Based on this assumption, 3D leaf edges were reconstructed by extracting the 2D leaf edges from true mask images and adopting a curve-based MVS reconstruction (Fig. 4, upper row); the reconstructed leaf edges appeared along the edges. Notably, the support threshold τ_t_ strongly affected the performance of curve-based reconstruction; low τ_t_ values resulted in highly inaccurate 3D curve fragments, and high values resulted in 3D curve fragments that did not completely cover the leaf edges (Fig. S3). Details regarding τ_t_ adjustment are discussed later (see Generation of mask images from actual multiview images using Mask R-CNN).

Regarding the 3D edges of multiple virtual leaves of a single plant, they were reconstructed after identifying the correspondences between individual leaves across the mask images, resembling reconstruction in the single-leaf case in all aspects except for considering the influence of occlusion (Fig. S4A). However, the correspondence of leaves among mask images is nontrivial in actual multiview images because the mask image is estimated for each individual image. Thus, we precisely estimated the 3D leaf edges of multiple leaves in a single scene by incorporating a leaf correspondence identification step that prevented the generation of pair hypotheses between noncorresponding leaves across views (Fig. 4, lower row, and Movie S1). In the absence of leaf correspondence identification, the number of reconstructed curve fragments decreased, and the vertical reconstruction error increased (Fig. S4B).

### Accuracy of 3D leaf edge reconstruction under different conditions

**Fig. 5.**
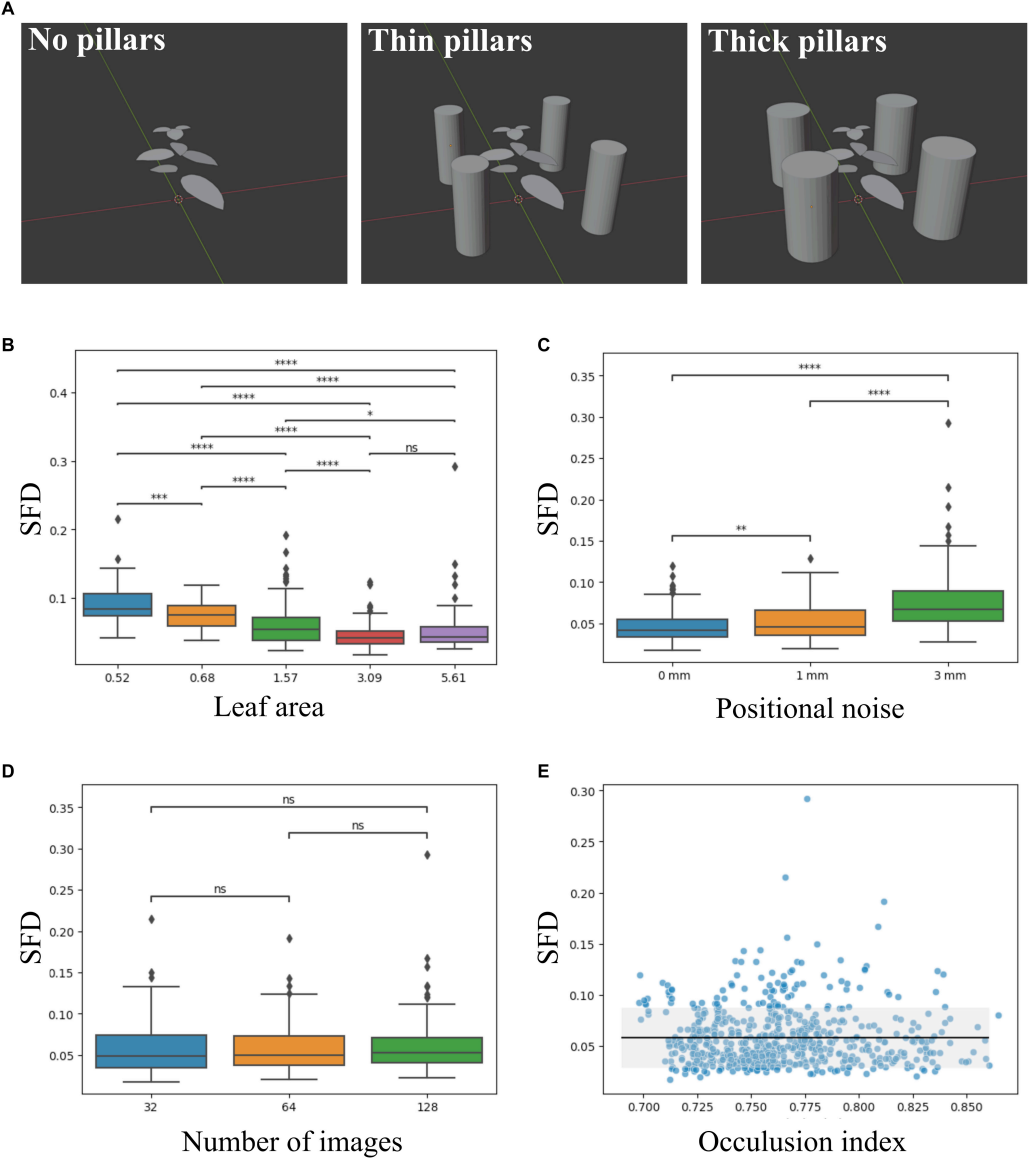
Simulations for evaluating the accuracy of 3D reconstruction. (A) Three levels of occlusions are assumed: no pillars (left), thin pillars (middle), and thick pillars (right). Box plots of SFD for leaf area (B), positional noise (C), and the number of images (D). Asterisks indicate significant differences between groups (pairwise Mann–Whitney U tests, ns: *P* ≥ 0.05, *: *P* < 0.05, **P < 0.01, ***P < 0.001, ****P < 0.0001). (E) Scatter diagram of SFD against OI. The predictive distribution was estimated using Bayesian ridge regression (black line: mean, light gray region: mean ± SD) on the simulated data (blue dots).

We evaluated the accuracy of the 3D leaf edge reconstruction method for different leaf areas, image numbers, occlusion levels, and noise levels, using dataset_s1_ (Fig. 5 and Fig. S5). The SFD decreased with the increase in leaf area; the small leaves were more challenging to reconstruct than the larger leaves were (Fig. 5B). Small leaves had larger curvatures even if they had the same shapes, making it difficult for the curve-based MVS approach to reconstruct the correct curve fragments because 2D curve fragments had been frequently generated through splitting by a tangential epipolar line (see Curve-based 3D reconstruction for details). The SFD increased with increases in the degree of noise at the camera positions. Although a less accurate camera extrinsic parameter estimation would increase the SFD, the effect might be limited under low noise (Fig. 5C). However, the SFD was less sensitive to the number of images and level of occlusion (Fig. 5D and E), considering that even if a leaf edge was obscured in an image, it could be complemented if it had appeared in other images [62].

### Guidelines for setting support thresholds in 3D edge reconstruction

**Fig. 6.**
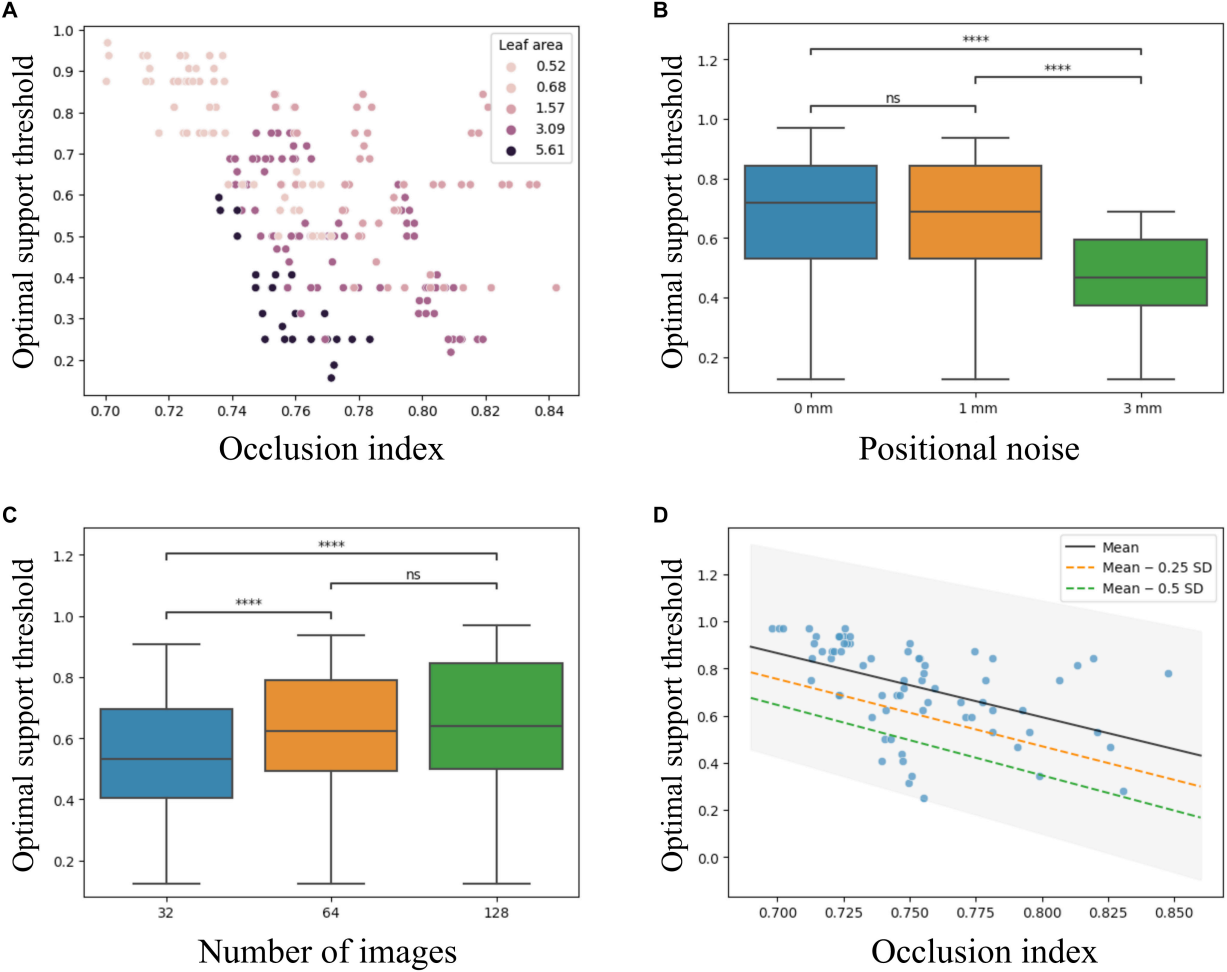
Optimal support thresholds proposed based on the simulated leaf data. (A) Scatter diagram of optimal support thresholds against the OI. Each point corresponds to the optimal support threshold that achieves the largest recall when the precision is greater than 0.99. The colors of the markers indicate the leaf area. Box plots of optimal support thresholds for (B) camera positional noise and (C) multiview images. Asterisks indicate significant differences between groups (pairwise Mann–Whitney U tests, ns: *P* ≥ 0.05, *: *P* < 0.05, **P < 0.01, ***P < 0.001, ****P < 0.0001). (D) Line plot of the mean (black line), the mean − 0.25 SD (orange dashed line), and the mean − 0.5 SD (green dashed line) of the predictive distribution of Bayesian ridge regression on the optimal support thresholds against the OI. The predictive distribution was estimated on the simulated data of 128 images with no camera positional noise (blue dots). The light gray region corresponds to the range of the mean ± SD.

The optimal support threshold increased for less occluded leaves (OI < 0.75), which had appeared in many images, because they had achieved both high precision and recall values by filtering inaccurate 3D curve fragments (Fig. 6A). Highly occluded leaves (OI > 0.75) tended to have lower optimal support thresholds at increased OI values, with the optimal values exhibiting large variations, which were attributed to differences in leaf areas, with larger leaves showing steeper trends. Furthermore, the optimal support thresholds decreased when the degree of camera positional error increased (i.e., low positional accuracies prevented precise filtering) (Fig. 6B) and increased slightly when there were more cameras (Fig. 6C). These trends were observed clearly in leaves with low to intermediate levels of occlusions (0.75 to 0.80) (Fig. S6).

Herein, we propose a guideline for determining the support threshold based on simulated data. We conducted Bayesian ridge regression on the optimal support thresholds against the OI based on the simulated data of 128 images with no camera positional noise (Fig. 6D). Moreover, we have provided the following qualitative guidelines: the slope of the linear regression models should be made a downward revision for large leaves (i.e., the trend becomes steeper when the leaf area becomes larger) (Fig. 6A); the camera positional error should be suppressed under a certain value (Fig. 6B); and the number of images should not be increased unnecessarily because improvements in estimation precision reduce when there are more images (Fig. 6C).

### Generation of mask images from actual multiview images using Mask R-CNN

**Fig. 7.**
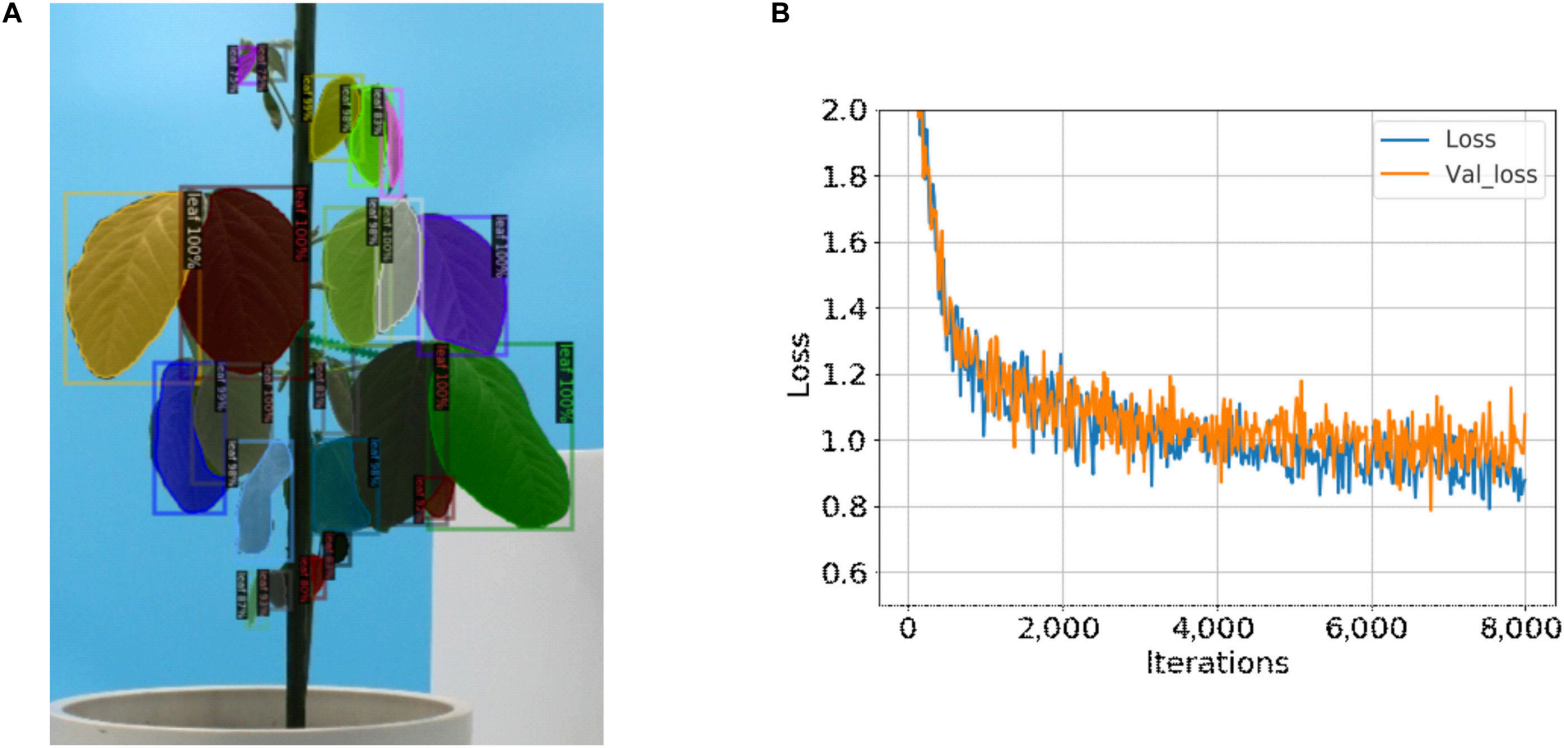
Mask image generation using Mask R-CNN. (A) Example of predicted masks of leaves. (B) Line plot representing the losses of Mask R-CNN. The validation loss became constant after ca. 7,000 epochs.

To generate a mask image for each leaf from multiview images of actual plants, we used Mask R-CNN [48], which is a DNN model used for instance segmentation. The performance of the model was evaluated on dataset_a1_ (the Confirmation of the proposed method on actual soybean data). The model weights were adopted at epoch 8,000, because the validation loss did not improve thereafter on the learning curve until epoch 10,000 (Fig. S7). Individual leaf masks were generated using the trained model (Fig. 7A). The average precision (AP) values of the test data are listed in Table. Regarding the values, AP was 49.8, and AP large (APl) was 76.9, indicating that inference had been successful in a large region, resembling the trend in a previous study on generic object recognition [48]. On the other hand, AP middle (APm) and AP small (APs) were smaller than APl, suggesting that generating masks for small leaves had been challenging.

**Table.** AP values of Mask R-CNN. Evaluated AP, AP50, AP75, APs, APm, and APl values on 4 individuals (Enrei, Zairai 51-2, Aoakimame, Saga zairai) as the test dataset.

	AP	AP50	AP75	APs	APm	APl
Enrei	30.8	42.3	33.0	0.7	8.1	65.2
Zairai 51-2	57.2	71.7	61.5	0.2	33.1	83.9
Aoakimame	45.1	58.9	48.2	1.0	30.6	78.3
Saga zairai	66.1	81.7	71.4	2.2	27.7	80.2
Average	49.8	63.6	53.5	1.0	24.9	76.9

After evaluating the performance of the instance segmentation model, the model was trained on all images of the 4 individuals until epoch 8,000 (Fig. 7B). We adopted the trained model for the analysis of actual soybean data (Application of proposed method to actual soybean data).

### Application of proposed method to actual soybean data

**Fig. 8.**
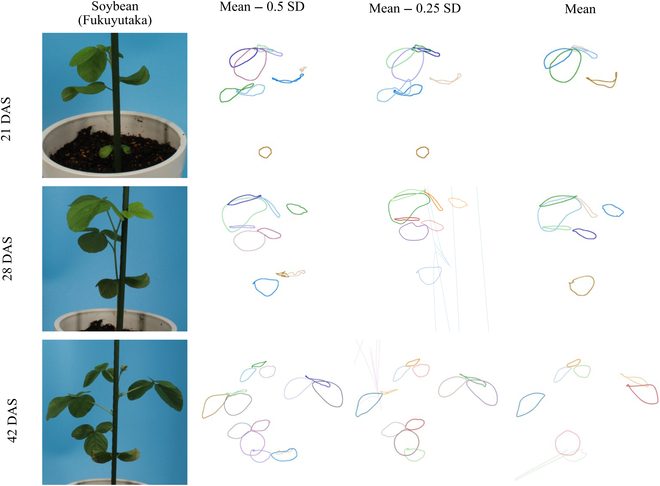
Reconstructed 3D leaf edges of actual soybean plants. Each row corresponds to a different growth stage (21, 28, and 42 DAS). Examples of a part of the 2D image of the multiview images are shown in the left column. Results of 3D leaf edge reconstructions with different support thresholds are shown in the right 3 columns: the mean − 0.5 SD, the mean − 0.25 SD, and the mean of the predictive distribution of the optimal support threshold to the OI of each leaf. Several failed cases are observed (see Fig. 9 for details).

**Fig. 9.**
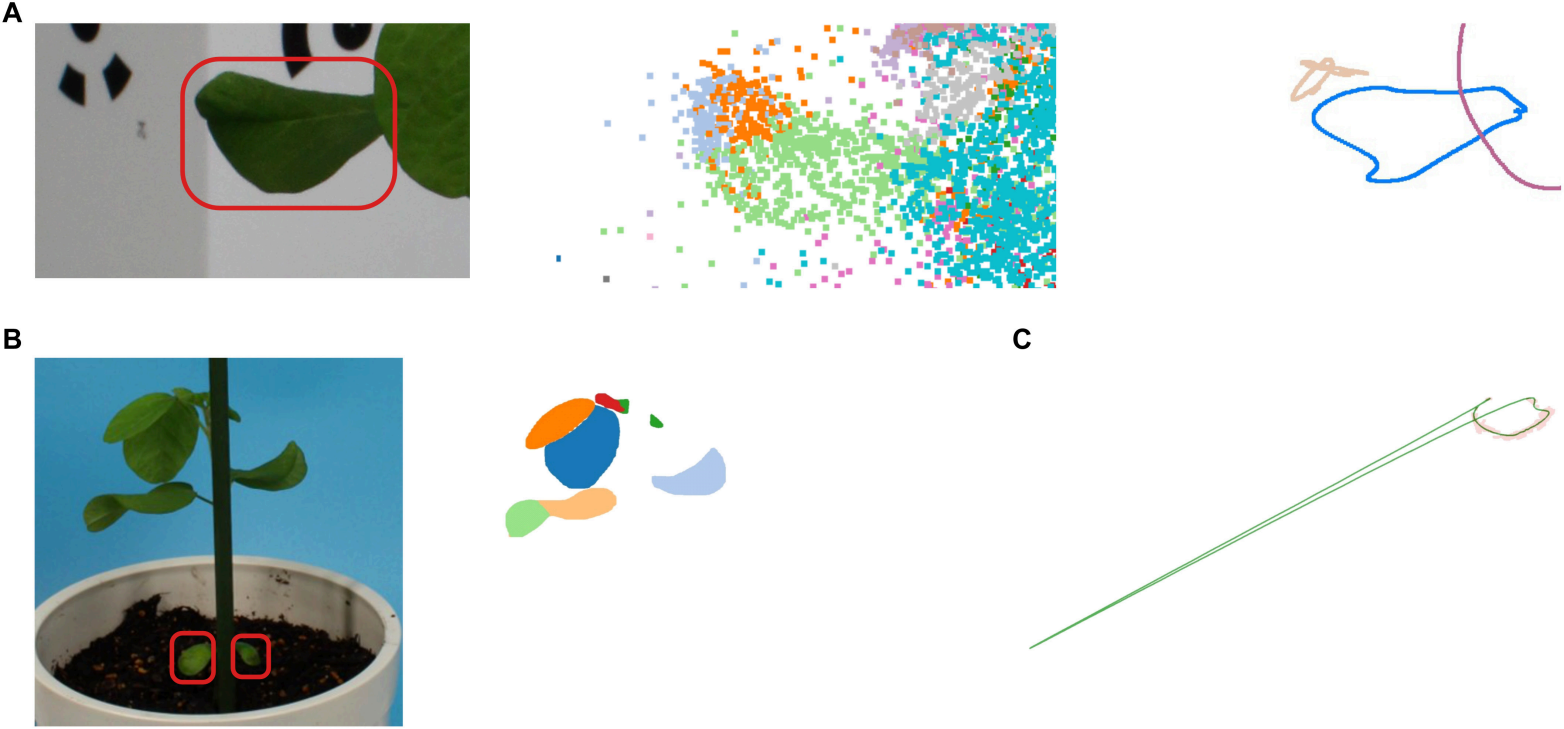
Three typical failed cases of 3D leaf edge reconstructions. (A) Single leaf reconstructed as multiple leaves. Although a single leaf in the 2D image (right) is observed, the point cloud data of the leaf has been segmented into multiple clusters (middle). Then, 2 edges are reconstructed based on the leaf (right; blue and beige edges). (B) Leaves have not been reconstructed. A pair of cotyledons are observed in the 2D image (left). They have not been reconstructed because Mask R-CNN has failed to predict them (right). (C) Leaf edge that has been reconstructed far from the actual position. When the 3D curve fragments are not covered over the leaf edge (pink lines: 3D curve fragments), the B-spline curve is overfitted to the boundaries (green line: estimated B-spline curve).

We demonstrated the performance of the proposed method on the actual multiview images by applying it to dataset_a2_, including individual soybeans at 3 growth stages (Fig. 8 and Movies S2 to S4). At the support threshold following the guidelines, inaccurate reconstructions were suppressed, but some leaves disappeared (Fig. 8, mean). At the lower support threshold than the proposed values, there was inaccurate reconstruction and the occurrence of artifacts, but almost all the leaves had been reconstructed (Fig. 8, mean − 0.5 SD). It was more challenging to reconstruct all the leaves at a later growth stage because of higher occlusions caused by increasing the number of leaves. Several types of failure cases were observed: (a) single leaves were reconstructed as multiple leaves because point cloud segmentation had failed in the leaf correspondence step (Fig. 9A); (b) leaves were not reconstructed because the small leaves had disappeared at the mask generation step (Fig. 9B); and (c) reconstructed leaf edges differed markedly from their original shapes owing to B-spline fitting in the cases where they had not been covered by 3D curve fragments (Fig. 9C).

### Applicability and limitations of the proposed method to different types of leaves

To describe the generalizability of the proposed method, it was applied to dataset_s2_, which featured complex leaf morphologies.

**Fig. 10.**
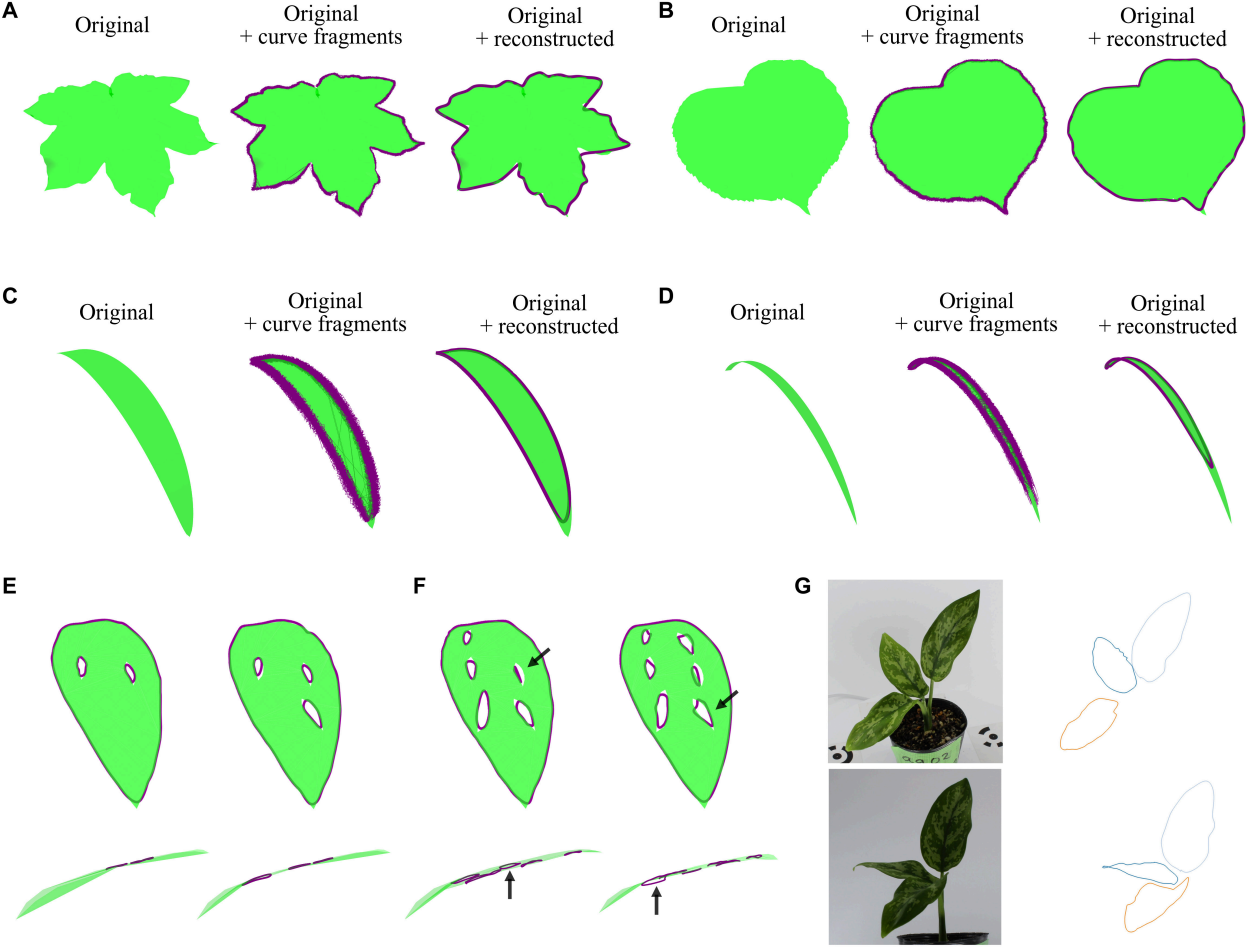
Leaf edge reconstruction of complex morphologies. (A) A lobed leaf. The mesh data (green) was adapted from “Maple Leaf” by Ciminera [56], used under CC BY 4.0 (cropped from the original mesh data). (B) A leaf with serration. The mesh data (green) was adapted from “Leaf test” by Ivanovs [57], used under CC BY 4.0 (cropped from the original mesh data). Elongated leaves. Leaves with an aspect ratio of 0.16 (C) and 0.04 (D). (E and F) Leaves with holes. For leaves with 3 or fewer holes, the edges and holes were accurately reconstructed (E). The accuracy of the reconstruction decreased when the number of holes increased to 5 and 6 (F). The ground-truth meshes (green regions). The reconstructed 3D curve fragments (purple curves) (middle of [A] to [D]). The reconstructed leaf edges (purple closed curves). (G) The reconstructed 3D leaf edges of *Aglaonema* ‘Maria’ (support threshold: mean − 0.5 SD).

The 3D leaf edge of the lobed leaf was reconstructed using the proposed method, except for the deepest parts of the indentation (Fig. 10A). Most of the 3D curve fragments were accurately reconstructed along the leaf's edge, including the most pronounced indentations; it was observed that the unsuccessful parts were attributable to the inadequate placement of knots in the B-spline curve fitting.

However, although the overall outline of serrated leaves was captured, the proposed method did not achieve the detailed reconstruction of each tooth in the serration (Fig. 10B). This was due to the generation of short curve pairs that were not adequately filtered out, resulting in an averaged reconstruction that lacked the serration details.

For elongated leaves, the edges were reconstructed, excluding the apex (Fig. 10C). Near the apex, there was a reduction in the number of reconstructed curve fragments, leading to the fitting of the B-spline curve predominantly in regions further from the apex. This problem became worse with an increase in the aspect ratio of the leaves, which correspondingly led to reduced accuracy in the reconstruction of the apex (Fig. 10D).

For leaves with 3 or fewer holes, the edges and holes were well reconstructed using the proposed method (Fig. 10E). However, as the number of holes increased, the precision of the reconstruction diminished. Especially for leaves with 5 and 6 holes, the holes appeared perpendicular owing to decreased accuracy of the 3D curve fragment reconstruction (Fig. 10F).

All 3D leaf edges of *Aglaonema* ‘Maria’ were reconstructed using manually created mask images (Fig. 10G). Similar to other cases, the leaf apex exhibited slight chipping but was successfully reconstructed, capturing the 3D undulation of the leaf edges.

## Discussion

The proposed phenotyping approach, which includes instance segmentation of 2D images and curve-based 3D reconstruction that integrates the information into a 3D space, successfully reconstructed 3D leaf edges from multiview images of both virtual and actual plants (Figs. 4 and 8). The proposed method was available to reconstruct 3D leaf edges with complex shapes, achieving a degree of success in reconstructing features such as the lobed leaf (Fig. 10A) and leaf holes (Fig. 10C). Thus, we will be able to address the morphological characteristics of 3D leaf edges, which have been difficult to evaluate quantitatively. However, it was still challenging when dealing with morphologies exhibiting highly local variations, like serrations (Fig. 10B) and leaf tips (especially in elongated leaves; Fig. 10C and D). Owing to the inclusion of the leaf correspondence identification step, our approach is applicable not only to a single leaf but also to multiple leaves in the same scene (Fig. 4). The direct 3D reconstruction of leaf edges does not require the removal of artifacts from the background and allows the robust estimation of leaf edges in a 1-dimensional closed curve in 3D space. The simulation results showed that as long as the camera positional errors were not too large (~1 mm), the precision in estimating the leaf edges could be maintained (Fig. 5), even when the number of cameras had been reduced or the degree of occlusion had been changed. Considering these results, although the proposed method works for individual plants with multiple leaves, further developments are required to apply it to major crops in dense canopies, which tend to have high occlusion and under field conditions where it is challenging to reduce camera positional errors (e.g., [32–35]). Moreover, the proposed method paves the way for solving the problem of point-based 3D reconstruction methods such as SfM/MVS, which are struggling to distinguish real holes from artifacts (e.g., [47]). The proposed method correctly performs 3D reconstruction only for the holes in leaves recognized in 2D images instead of incorrectly recognizing these holes as the “negative” of the point cloud data. In our simulation, the holes were reconstructed well when the number of holes was less than 4 (Fig. 10E). Although the estimation was poor when the number of holes was greater than 4, the results would be improved by recognizing each hole as an individual instance in the instance segmentation step, similar to the approach in leaf correspondence identification (Fig. S4).

To improve the accuracy of 3D edge reconstruction, the following points should be considered: (a) tuning the hyperparameters, (b) improving the camera parameter estimation, and (c) improving the instance segmentation model. These points are elaborated as follows: (a) The hyperparameters used in the proposed method were tuned. We provided the guidelines for setting the support thresholds (*τ_t_*) against the target leaf area (*A*), OI, the degree of positional error (*σ*), and the number of images (Fig. 6); however, other parameters also played crucial roles in 3D edge reconstruction. For example, the fragment length potentially played a primary role in improving the accuracy of small leaves. In this study, we used a fragmentation length appropriate for larger structures ((*l*_min_, *l*_max_) = (40, 100) and (*l*_min_, *l*_max_) = (80,200) for the simulation and actual data, respectively) in the 2D edge extraction, which reduced the number of 2D curve fragments for smaller structures. In postprocessing using B-spline curve fitting, the number of knots should be tuned to capture high-curvature leaf edges (e.g., [63]). This will be critically important in reconstructing complex leaf edge shapes, such as lobed leaves (Fig. 10A). (b) Accurate camera parameter estimation improved the accuracy of 3D reconstruction. We robustly estimated camera parameters in SfM using coded and noncoded markers. A curve-based bundle adjustment for camera parameter calibration by minimizing the curve-based reprojection error, used by Fabbri and Kimia [50], could lead to accuracy enhancements. (c) Improving the AP values of the instance segmentation model improved the performance of 3D reconstruction (Fig. 9B). The Mask R-CNN model trained on our dataset showed that APs that had been considered to account for most of the mask generation accuracy of small leaves were smaller than APl and APm and were unsuitable for reconstructing immature leaves (Table 1). Therefore, it is desirable to expand the dataset, especially for small leaves. The use of pretraining models with large datasets, such as the segment anything model [64], is also promising for generating high-quality mask images for each instance, especially when applying to leaves exhibiting diverse morphologies and textures. Alternatively, a model capable of directly recognizing anatomical structures of interest in plants may be useful (e.g., [65,66]).

In this study, we proposed guidelines for setting the support threshold when applying the proposed method to actual plants. These guidelines mainly depend on the level of occlusion and noise and the number of images (Fig. 7). We investigated the advantages of the curve-based approach, learning that a limited number of images were sufficient for estimating 3D leaf edges. Reconstruction was successfully performed following these guidelines and verified using actual individual soybean data (Fig. 9). The guidelines helped us determine the configuration of the experimental designs and data acquisition scenarios, including the hyperparameters.

The proposed method is an essential technique for assessing the 3D morphological properties of leaves, which are challenging to quantitatively evaluate. These properties play a central role in balancing the multiple functional demands of individual plants and canopies [5–7], with traditional evaluations mostly being performed using 2D approaches (e.g., [21,22]). The proposed method obtained 3D leaf edges, including their 3D positions, orientations, and sizes, relative to the configurations of organs in individual plants in a nondestructive manner (Fig. 9). It is a promising method to capture whole-plant architecture combined with a method for estimating branches [67,68], other plant organs [69,70], and leaf anatomical structures including leaf veins [71,72], textures, and holes. Furthermore, FSPMs, which couple the 3D morphologies of plants with their physiological dynamics, can be improved and validated using morphological data obtained using the proposed method and their morphometric features (e.g., 3D elliptic Fourier descriptors). For example, the optimal morphologies and movements (e.g., optimal canopy structure [37], diurnal leaf movement [73], and leaf phototropism [74]) predicted using FSPMs in previous studies were tested to determine how they fit the experimental data and vice versa. Our proposed method contributes to filling this gap by successfully integrating hierarchical morphological properties into 3D spaces.

## Data Availability

The datasets used and/or analyzed during the current study are available in the repositories on Zenodo (10.5281/zenodo.10836254, 10.5281/zenodo.10836258, 10.5281/zenodo.10836260, 10.5281/zenodo.10065546, 10.5281/zenodo.10828962, 10.5281/zenodo.10121073, and 10.5281/zenodo.10829007) and GitHub (https://github.com/MorphometricsGroup/Murata-2024).
