# Supplementary material for: Three-Dimensional Leaf Edge Reconstruction Combining Two- and Three-Dimensional Approaches
Source: Plant Phenomics. 2024 May 9;6:0181. doi: 10.34133/plantphenomics.0181 (PMC11079596; doi:10.34133/plantphenomics.0181)
Supplement: Supplementary 1 — Figs. S1 to S7 Movies S1 to S4 [file plantphenomics.0181.f1.zip › supplementary_materials.pdf]

Supplementary Materials

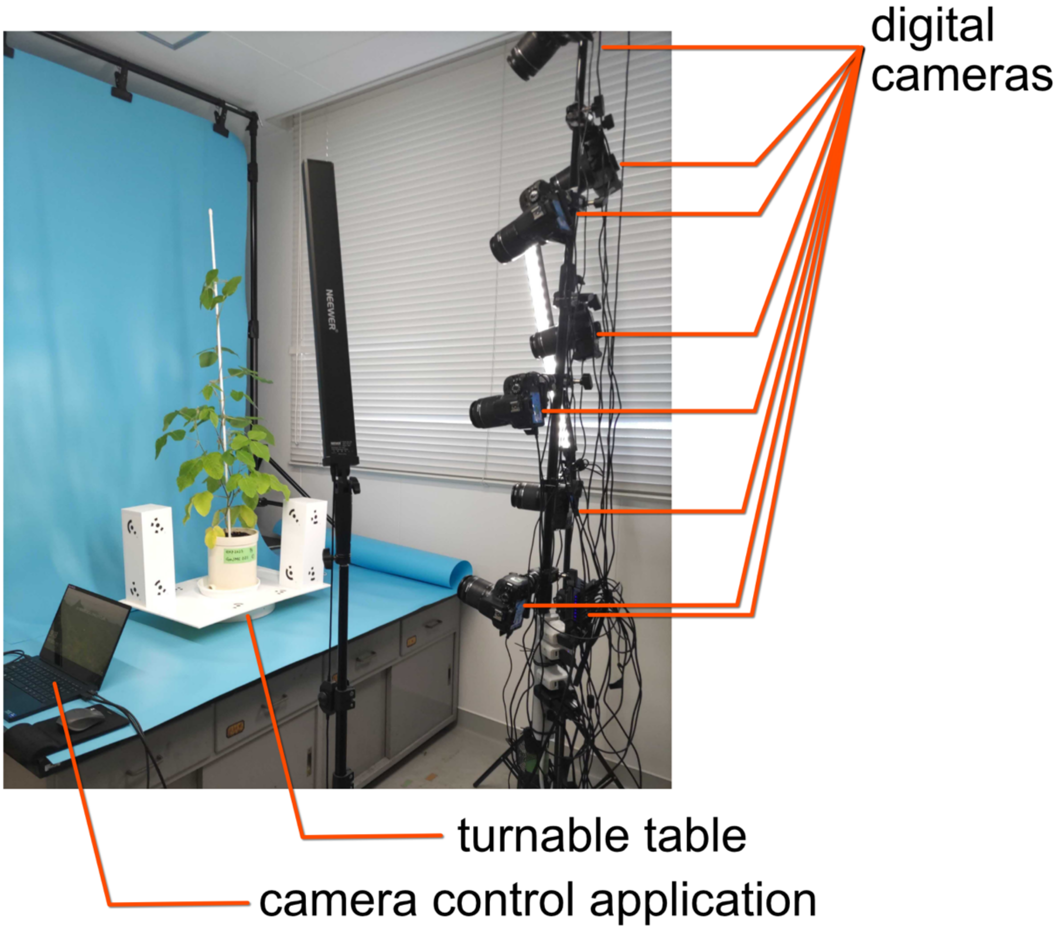

Figure S1. Photogrammetry system. A soybean individual is placed on a turntable table. Multi-view images of the plant have been taken by 8 digital cameras controlled by digital photography software.

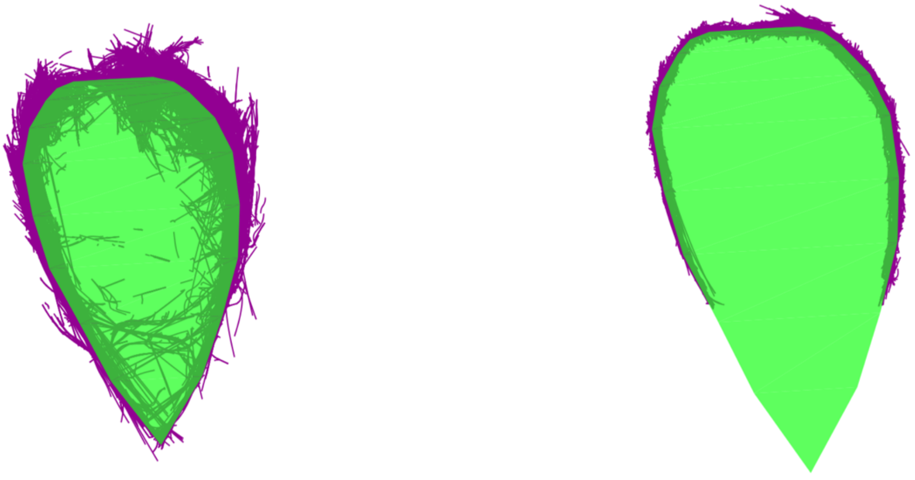

Figure S2. Support threshold balances the number of images and the accuracy of reconstruction. More 3D curve fragments, including inaccurate ones, have been reconstructed with a lower support threshold (left). Less, accurate 3D curve fragments with a higher support threshold have been reconstructed (right).

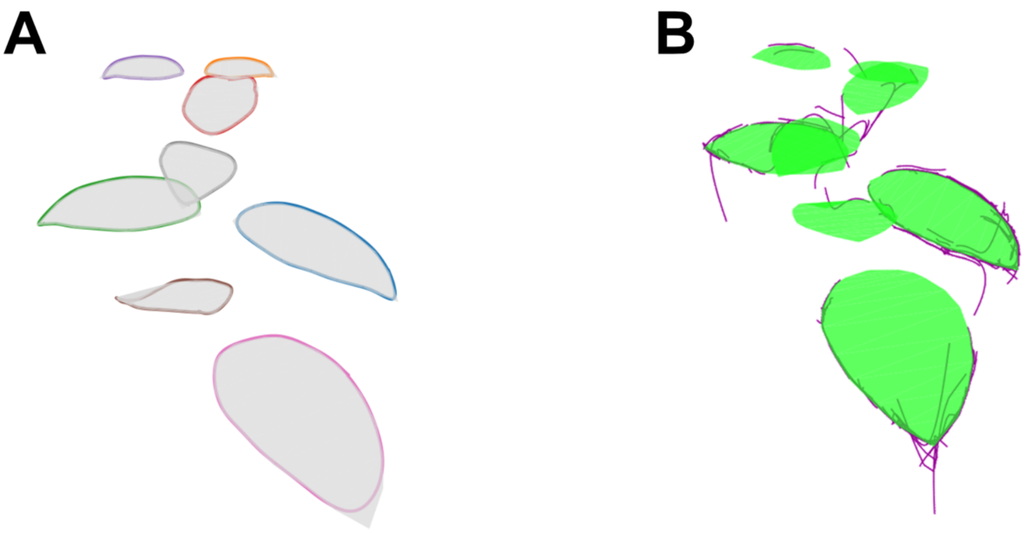

Fig. S3. Leaf correspondence on multiple leaves in the same scene. T The 3D leaf edges are reconstructed if the leaf correspondences are known (left). Reconstructed leaf edges (curves with different colors) are reconstructed along the edges of the original mesh (gray). However, the 3D leaf edge reconstruction fails when the leaf correspondences are not known, and they are attempted to be simultaneously reconstructed (right). Purple curves indicate 3D curve fragments.

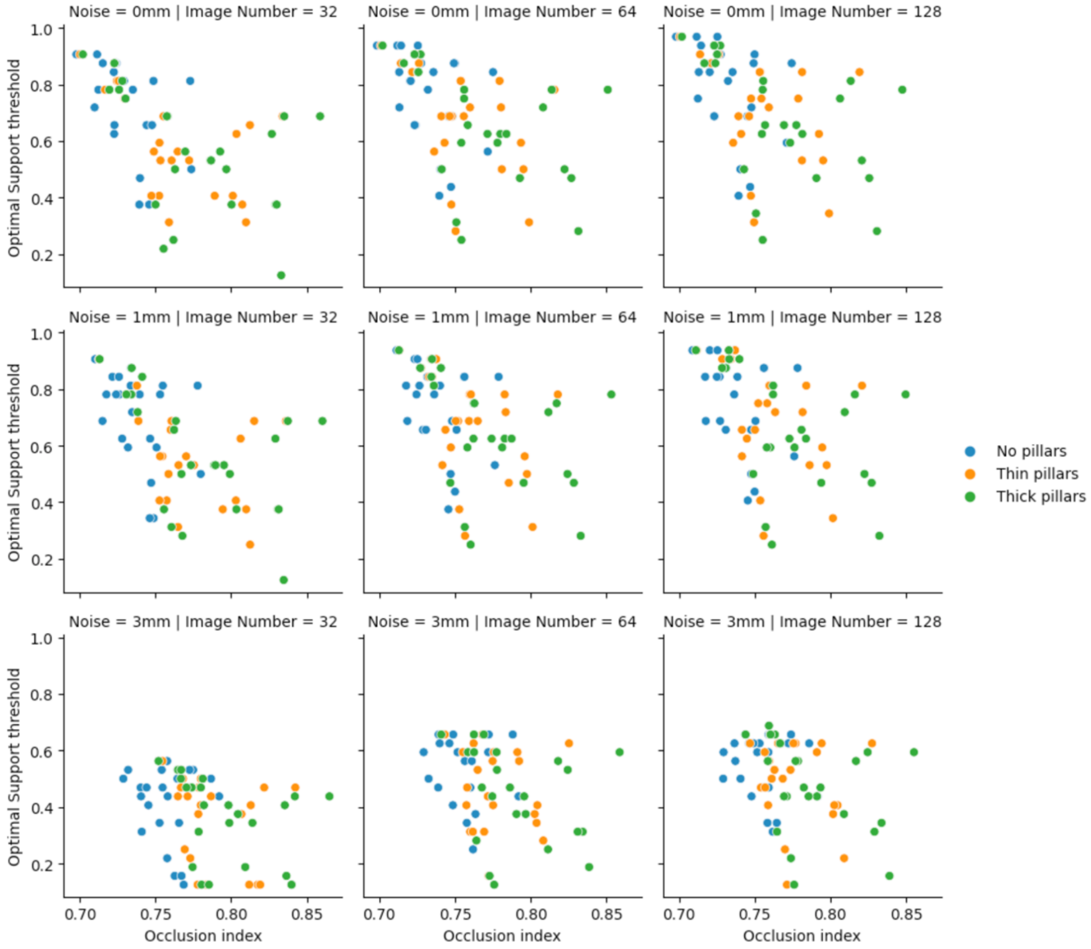

Figure S4. Scatter diagrams of the optimal support thresholds. Each diagram shows the optimal support thresholds to the OI with different values for camera positional noise (row) and the number of images (column).

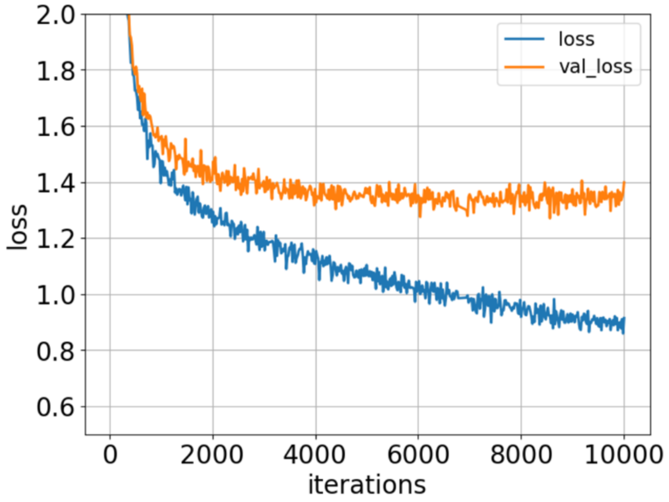

Figure S5. Learning curve of Mask R-CNN using Enrei as test data.

Movie S1. The reconstructed 3D leaf edges of the simulation data.

Movie S2. The reconstructed 3D leaf edges of the actual soybean plant (Fukuyutaka, 21 DAS, mean – 0.25 SD).

Movie S3. The he reconstructed 3D leaf edges of the actual soybean plant (Fukuyutaka, 28 DAS, mean – 0.5 SD).

Movie S4. The reconstructed 3D leaf edges of the actual soybean plant (Fukuyutaka, 42 DAS, mean – 0.5 SD).
